# Chemoprevention of LA7-Induced Mammary Tumor Growth by SM6Met, a Well-Characterized *Cyclopia* Extract

**DOI:** 10.3389/fphar.2018.00650

**Published:** 2018-06-20

**Authors:** Omolola R. Oyenihi, Annadie Krygsman, Nicolette Verhoog, Dalene de Beer, Michael J. Saayman, Thys M. Mouton, Ann Louw

**Affiliations:** ^1^Department of Biochemistry, Stellenbosch University, Stellenbosch, South Africa; ^2^Department of Physiological Sciences, Stellenbosch University, Stellenbosch, South Africa; ^3^Post-Harvest and Agro-Processing Technologies, Agricultural Research Council of South Africa, Infruitec-Nietvoorbij, Stellenbosch, South Africa; ^4^Department of Food Science, Stellenbosch University, Stellenbosch, South Africa; ^5^Department of Biomedical Sciences, Cape Peninsula University of Technology, Cape Town, South Africa

**Keywords:** chemoprevention, *Cyclopia*, mammary tumor, phytoestrogen, tamoxifen, letrozole, fulvestrant

## Abstract

Breast cancer (BC) is the leading cause of cancer-related deaths in women. Chemoprevention of BC by using plant extracts is gaining attention. SM6Met, a well-characterized extract of *Cyclopia subternata* with reported selective estrogen receptor subtype activity, has shown tumor suppressive effects in a chemically induced BC model in rats, which is known to be estrogen responsive. However, there is no information on the estrogen sensitivity of the relatively new orthotopic model of LA7 cell-induced mammary tumors. In the present study, the potential chemopreventative and side-effect profile of SM6Met on LA7 cell-induced tumor growth was evaluated, as was the effects of 17β-estradiol and standard-of-care (SOC) endocrine therapies, such as tamoxifen (TAM), letrozole (LET), and fulvestrant (FUL). Tumor growth was observed in the tumor-vehicle control group until day 10 post tumor induction, which declined afterward on days 12–14. SM6Met suppressed tumor growth to the same extent as TAM, while LET, but not FUL, also showed substantial anti-tumor effects. Short-term 17β-estradiol treatment reduced tumor volume on days prior to day 10, whereas tumor promoting effects were observed during long-term treatment, which was especially evident at later time points. Marked elevation in serum markers of liver injury, which was further supported by histological evaluation, was observed in the vehicle-treated tumor control, TAM, LET, and long-term 17β-estradiol treatment groups. Alterations in the lipid profiles were also observed in the 17β-estradiol treatment groups. In contrast, SM6Met did not augment the increase in serum levels of liver injury biomarkers caused by tumor induction and no effect was observed on lipid profiles. In summary, the results from the current study demonstrate the chemopreventative effect of SM6Met on mammary tumor growth, which was comparable to that of TAM, without eliciting the negative side-effects observed with this SOC endocrine therapy. Furthermore, the results of this study also showed some responsiveness of LA7-induced tumors to estrogen and SOC endocrine therapies. Thus, this model may be useful in evaluating potential endocrine therapies for hormone responsive BC.

## Introduction

Breast cancer (BC) is the most commonly diagnosed malignancy in women worldwide and a leading cause of cancer-related death in women. Approximately 80% of all diagnosed BCs express the estrogen receptor (ER) and are dependent on estrogen for proliferation (Lumachi et al., 2015). Estrogen stimulates the growth of BC through ER-mediated mechanisms and formation of genotoxic estrogen metabolites (Santen et al., 2015).

The goal of antihormonal therapy is to reduce ER signaling by directly antagonizing or downregulating ER or indirectly by reducing estrogen levels via inhibition of aromatase, a key enzyme in estrogen synthesis. Standard-of-care (SOC) endocrine therapies such as tamoxifen (TAM), a selective ER modulator (SERM), and fulvestrant (FUL), a selective ER down-regulator (SERD), interfere with ER signaling *via* direct effects on the ER. TAM acts by competitively antagonizing estrogen binding to the ER in the breast, while FUL accelerates ER degradation thereby reducing cellular ERα levels (Nathan and Schmid, 2017). Alternatively, letrozole (LET), an aromatase inhibitor (AI), indirectly disrupts ER signaling by blocking the conversion of adrenal androgens to estrogen in non-ovarian tissues (Fabian, 2007).

Tamoxifen is extensively used as first line endocrine therapy in both pre- and post-menopausal women with hormone responsive (ER^+^) BC (Dixon, 2014). AIs are used as a monotherapy in post-menopausal women either as first or second line interventions (Wong and Ellis, 2004), while in pre-menopausal women with functional ovaries, AIs are used in conjunction with ovarian suppression/ablation (Fabian, 2007). FUL on the other hand is mostly used in the treatment of tumors that have become refractory to TAM or LET (Lumachi et al., 2015). Although these adjuvant endocrine options are still the mainstay for the treatment of ER-positive BC, *de novo* or acquired resistance (30–40% in patients receiving adjuvant TAM therapy) and associated side-effects (such as endometrial cancer, myocardial infarction, hepatic injury, and renal dysfunction) limit the clinical usefulness of these drugs (Hirsimäki et al., 2002; Kalender et al., 2007; Puhalla et al., 2012; Yang et al., 2013; Gao et al., 2016).

Despite the advances in BC treatment, prevention if possible is always better than treatment. Two SERMs, TAM and raloxifene, have been approved by the FDA for BC chemoprevention, although resistance and side-effects remain a huge challenge. Hepatic injury is one of the most severe side-effects of long-term use of TAM (Yang et al., 2013). There is a growing interest in the use of natural compounds, specifically phytoestrogens (plant-derived estrogen-like molecules), as potential chemopreventative agents in mammary carcinogenesis (Mense et al., 2008; Kado et al., 2012; Hwang and Choi, 2015). The consumption of phytoestrogens is associated with a reduced incidence of pre-menopausal BC in East Asian nations compared to the Western World (Adlercreutz, 2002). Paradoxically, unlike estrogen, phytoestrogens have demonstrated protective effects in BC via multi-targeted actions such as: weak estrogenicity, reduction in local estrogen production, antiproliferative and antioxidant activities, epigenetic modifications and topoisomerase inhibition, among others (Bolego et al., 2003; Rice and Whitehead, 2006). In addition, low cytotoxicity to patients and lack of side-effects in clinical trials have stimulated interest in the investigation of the anticarcinogenic effects of phytoestrogens (Virk-Baker et al., 2010).

*Cyclopia* (Family: Fabaceae) is a fynbos plant used as an aromatic herbal tea called honeybush tea (Du Toit et al., 1998). There are over 20 *Cyclopia* species indigenous to the Eastern and Western Cape regions of South Africa (Schutte, 1997). Some documented beneficial properties of *Cyclopia* include antimutagenic (Van der Merwe et al., 2006), antioxidant (Marnewick et al., 2003), anticancer (Marnewick et al., 2005, 2009; Sissing et al., 2011), and antidiabetic (Larsen et al., 2008) effects. In addition, there is documented evidence that *Cyclopia* is a source of phytoestrogens (Verhoog et al., 2007).

*Cyclopia subternata* is one of the species primarily used to produce honeybush tea. Many phenolic compounds with health promoting benefits have been identified in *C. subternata* (De Beer et al., 2012; Kokotkiewicz et al., 2012). SM6Met is a well-characterized phytoestrogenic extract of *C. subternata* with a comparable estrogenic potency to commercially available phytoestrogenic nutraceuticals (Mfenyana et al., 2008; Mortimer et al., 2015). Previous work from our group have shown that SM6Met antagonizes estrogen-induced BC cell proliferation *in vitro* (Visser et al., 2013). Although estrogen has a similar affinity for both ER subtypes, studies in MCF7 BC cells suggest that ERβ opposes the proliferative effects exerted by ERα (Ström et al., 2004; Koehler et al., 2005). The preferential affinity of many phytoestrogens for ERβ compared to ERα might thus be clinically relevant in BC. SM6Met, for example, has displayed ER subtype specific estrogenic activity namely, ERα antagonism and ERβ agonism (Visser et al., 2013). In an immature rat uterotrophic model, the lack of uterine growth and delayed vaginal opening following treatment with the SM6Met extract provides additional evidence for its anti-estrogenic and ERα antagonistic effect (Visser et al., 2013). In addition, the inhibitory effects of SM6Met on cell cycle progression have been documented (Visser et al., 2016).

LA7 is a mammary adenocarcinoma cell line isolated from 7,12-dimethylbenz[a]anthracene (DMBA)-induced BC rats (Zucchi et al., 2007). The induction of mammary tumors with LA7 cancer cells is a relatively new orthotopic method of developing solid mammary gland tumors in immunocompetent rats. The advantages of this model are the strong tumorigenic properties of the cells and a relatively short duration, within 7–10 days, of tumor development. This is a significantly shortened experimental protocol compared to the 8–10 weeks of tumor latency in chemically induced [methyl-*N*-nitrosourea (MNU) and DMBA] mammary carcinogenic models (Abbasalipourkabir et al., 2010; Karimian et al., 2015; Alvarado et al., 2017). Not only is the LA7 model a faster method of tumor induction, but it is also ideally suited for testing therapies specifically targeting late stage carcinogenesis (progression) while circumventing the earlier phases. Although the chemical carcinogen models (DMBA and MNU) permit analysis of the early stages of carcinogenesis (induction and promotion), they are also suitable for the study of malignant progression or the advanced stage of mammary cancers, however, the longer latency often required for development of tumors remain an important limitation. Thus, the present model is particularly useful for investigating suppressing agents that interfere with later stages of carcinogenesis (progression) by simulating the delay of the growth of existing occult breast tumors and as such may be termed chemoprevention (Santen et al., 2017). The short tumor latency of the LA7 model might also be advantageous for a quicker assessment of potential endocrine therapies for BC compared to the longer latency encountered in hormone-dependent MNU and DMBA models.

To date, there is no information regarding the estrogen responsiveness of LA7-induced mammary tumors. However, a few available studies have demonstrated the ability of TAM to suppress the growth of LA7 mammary tumors, suggesting the endocrine responsiveness of this model (Syam et al., 2016; Bakar et al., 2017). Although the presence of ERα is a well-established predictor of response to TAM treatment in BC (Droog et al., 2013; Rondón-Lagos et al., 2016), evidence also exists that some ERα-negative tumors benefit from adjuvant TAM treatment (Gruvberger-Saal et al., 2007; Manna and Holz, 2016; Rondón-Lagos et al., 2016).

Several phytochemicals with documented anti-cancer effects have been identified in SM6Met (Louw et al., 2013). A combinational approach, involving the use of more than one bioactive dietary compound in chemoprevention or cancer therapy, holds great promise to progress into clinical chemoprevention trials especially if its efficiency is accompanied with minimal side-effects (Saldanha and Tollefsbol, 2012).

A recent study from our group has shown that SM6Met, a selective ER subtype modulator (SERSM), delays the growth of mammary tumors in a chemical carcinogen (MNU) model that is particularly known to be estrogen dependent (Visser et al., 2016). Considering the relatively short tumor latency and lack of information regarding the estrogen responsiveness of LA7-induced mammary tumors, in the present study, we examined the dose-dependent chemopreventative effect of SM6Met, in this model of mammary tumorigenesis and compared effects to that of SOC endocrine therapies. In terms of toxicity, we also report for the first time the effect of SM6Met on liver and kidney function. Furthermore, this is the first study to report the effect of estrogen treatment and SOC endocrine therapies, such as FUL and LET, on tumor growth in this relatively new orthotopic model of mammary tumorigenesis.

## Materials and Methods

### Chemicals

All the solvents used for plant extraction and quantitative HPLC analysis were of analytical grade and supplied by Merck Millipore, S.A. Acetonitrile and acetic acid were supplied by Sigma-Aldrich (South Africa). Protocatechuic acid, *p*-coumaric acid, 3-β-D-glucopyranosyliriflophenone (IMG), hesperidin, mangiferin, and scolymoside were purchased from Sigma-Aldrich (South Africa). Eriocitrin was purchased from Extrasynthese (France) and vicenin-2 was purchased from Phytolab (Germany). Isomangiferin was purchased from Chemos GmbH (Germany). 3′,5′-Di-β-D-glucopyranosylphloretin (PDG) and 3-β-D-glucospyranosyl-4-β-D-glucopyranosyloxyiriflophenone (IDG) were isolated from *Cyclopia subternata* (unpublished results) and *Cyclopia genistoides* (Beelders et al., 2014), respectively. Castor oil, 17β-estradiol, and SOC endocrine drugs were purchased from Sigma-Aldrich. All reagents for the estimation of biochemical parameters were purchased from Horiba ABX Pentra (Montpellier, France).

### Preparation of Plant Extract

Dried plant material (leaves) of a *C. subternata* harvesting (M6; harvested on 30 March 2004 from a commercial plantation at Kanetberg farm near Barrydale, South Africa) was extracted according to a previously described procedure (Mortimer et al., 2015).

Briefly, 500 g finely pulverized, dried *C. subternata* leaves were defatted with dichloromethane (2 L) by stirred extraction over a period of 24 h. Following extraction, the plant material was filtered, and the residue was air-dried overnight in a fume cabinet at room temperature. This process was repeated four times. Next, the air-dried, defatted plant material was subjected to sequential extraction using three solvents (2 L each) in order of increasing polarity (ethyl acetate, ethanol, and methanol). The sequential extraction step in the sequence was performed three times for 3 h per step. Before a solvent change was made, the plant material was air-dried overnight in a fume cabinet at room temperature. The filtrates of the final methanol extraction step were retained, pooled, and the methanol extract evaporated under vacuum (Büchi Rotavap, Switzerland) at 40°C. A small quantity of deionized water was added to the evaporated extract and the extract was freeze-dried (Virtis Advantage Plus, United States). The freeze-dried extract was ground with a pestle and mortar to obtain a fine powder (60.2 g) that was stored in a desiccator, protected from light, at room temperature. Batches of SM6Met were prepared using the procedure described above and a mixture of these batches was used for animal treatment.

### Quantification of Phenolic Compounds in SM6Met Using Quantitative HPLC (qHPLC)

Stock solutions of SM6Met and standards were prepared in DMSO and frozen at -20°C until needed for analysis. Defrosted extract and standards were appropriately diluted with water for experimental analysis and ascorbic acid (Sigma-Aldrich) was added to a final concentration of 9 mg/mL. The mixtures were then filtered using Millex-HV syringe filters (Millipore, United States) with a 0.45 μm pore size.

Analyses were performed on an Agilent 1200 HPLC consisting of an in-line degasser, quaternary pump, autosampler, column oven, and diode-array detector (DAD), controlled by Chemstation software (Agilent Technologies, Santa Clara, CA, United States). Separation was achieved on a Gemini-NX C_18_ column (150 × 4.6 mm; 3 μm; 110 Å; Phenomenex, United States), protected by a guard column (4 × 3.0 mm; Phenomenex) of the same stationary phase, with 2% aqueous acetic acid (A) and acetonitrile (B) as mobile phases. Separation was performed using the HPLC method described by De Beer et al. (2012). The column temperature was controlled at 30°C. A flow rate of 1 mL/min was used with the following mobile phase gradient: 0–2 (8% B), 2–27 (8–38% B), 27–28 (38–50% B), 28–29 (50% B), 29–30 (50–8% B), and 30–40 min (8% B). The dihydrochalcones [PDG and 3-hydroxyphloretin-3′,5′-di-*C*-hexoside (HDH)], flavanones (eriocitrin and hesperidin), benzophenones (IMG and IDG), and benzoic acid (protocatechuic acid) were quantified at 288 nm, whereas the xanthones (mangiferin and isomangiferin), flavones (vicenin-2, scolymoside, and luteolin), and hydroxycinnamic acid (*p*-coumaric acid) were quantified at 320 nm.

A 7-point calibration curve was set up for all the available standards. Due to the unavailability of a reference standard, HDH were expressed in terms of PDG equivalents.

### LA7 Cell Culture and Mammary Tumor Induction

The rat mammary gland tumor cell line, LA7 (ATCC No CRL2283) was obtained from the American Type Culture Collection (ATCC, Manassas, VA, United States). LA7 cells were cultured in high glucose (4.5 g/L) Dulbecco’s modified eagle’s medium (DMEM) supplemented with 10% FCS, 100 IU/mL penicillin, and 100 μg/mL streptomycin (1% penicillin–streptomycin) (Sigma-Aldrich, South Africa) and maintained at 37°C in a humidified atmosphere of 5% CO_2_. Stock cultures of LA7 cells at Passage number (P#) 12 were seeded in 75 cm^2^ flasks (SPL Life Sciences) and allowed to multiply for 48 h. Thereafter, cells (P#13) were transferred into 175 cm^2^ flasks (Nest Biotechnology) at 80–90% confluency and maintained in high glucose DMEM supplemented with 10% FCS and 1% penicillin–streptomycin for 72–96 h. To harvest cells, trypsin-EDTA was added to detach cells and neutralized using phosphate-buffered saline (PBS; Sigma-Aldrich). Cells were immediately centrifuged at 100 ×*g* for 10 min at 4°C. For viability detection, the cells were stained with trypan blue and counted using a cell counter.

For tumor induction, LA7 cells (prepared as described above) at P#14 were re-suspended at a concentration of 18 × 10^6^ in 300 μL of PBS per rat and inoculated subcutaneously into the mammary fat pad (right flank) using a tuberculin syringe and 26G needle. Injection of cells was performed under isoflurane anesthesia and cells (P#14) were used within 70 min of preparation.

### Animals and Experimental Design

This study was carried out in accordance with the recommendations of the South African National Standards 10386: 2008, Research Ethics Committee: Animal Care and Use of Stellenbosch University. The protocol was approved by the Research Ethics Committee: Animal Care and Use of Stellenbosch University (Ethical Approval No: SU-ACUM14-00019).

**FIGURE 1:**
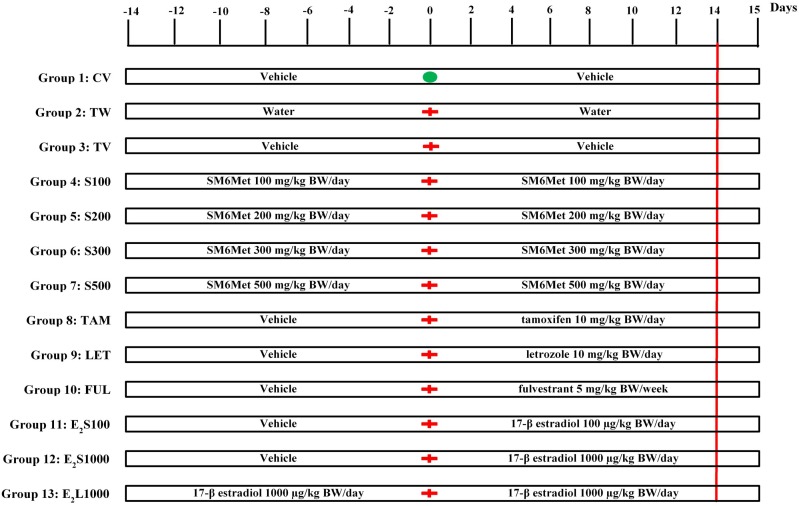
Experimental protocol. Animals were assigned into 13 different groups (groups 1–13). Treatment commenced 2 weeks prior to tumor induction on day-14. Tumor induction with LA7 cells occurred on day 0 (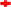) for all groups except group 1, which received a PBS injection (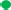). The treatment continued up to day 14 (red vertical line denotes end of treatment) and animals were euthanized on day 15. See text for further details.

Female Sprague-Dawley (SD) rats (180 ± 20 g, 49 days of age) were obtained from the Animal Facility of Stellenbosch University, South Africa. The animals were housed in propylene cages under standard conditions (temperature 22°C, relative humidity 40%, 12 h light–dark cycle). Animals were acclimatized for 1–3 weeks before the commencement of experimental procedures. Standard rodent pellets and tap water were accessible *ad libitum* to the animals. Rats were randomly divided into 13 groups as indicated below and summarized in **Figure 1**.

A mixture of ethanol and castor oil (ethanol:castor oil; 1:9) served as vehicle control. SM6Met, 17β-estradiol (E_2_), and SOC endocrine therapies (TAM, FUL, and LET) were dissolved/suspended in ethanol and diluted 10-fold with castor oil [1:9; 3 mL/kg body weight (BW)]. The doses of SM6Met (Visser et al., 2013, 2016), E_2_ (Kerdelhue and Jolette, 2002), TAM (Shamsabadi et al., 2013; Bakar et al., 2017), FUL (Osborne et al., 1995; Bean et al., 2015), and LET (Sinha et al., 1998) are similar to those previously published. All treatments were administered by oral gavage. Normal healthy rats in group 1 were treated daily with vehicle (CV) throughout the 28 days study duration. Groups 2 and 3 consisted of tumor-bearing rats treated daily with water (TW) or vehicle (TV), respectively, for 2 weeks prior to tumor induction and continued for 2 weeks thereafter. Groups 4–7 were pre-treated with increasing SM6Met (S) doses (100, 200, 300, and 500 mg/kg BW/day) for 2 weeks prior to tumor induction and treatment was continued for 2 weeks thereafter. Animals in groups 8, 9, and 10 were pre-treated with vehicle for 2 weeks before mammary tumor induction, after which treatment was switched to SOC endocrine therapies, TAM and LET (10 mg/kg BW/day), and FUL (5 mg/kg BW/week), respectively, and continued for 2 weeks. Animals in groups 11 and 12 were also initially treated with vehicle for 2 weeks prior to tumor induction and treatment was switched to E_2_ at 100 μg/kg BW/day (E_2_S100) and 1000 μg/kg BW/day (E_2_S1000), respectively, after tumor induction. Lastly, animals in group 13 (E_2_L1000) were pre-treated with 1000 μg/kg BW/day of E_2_ for 2 weeks prior to exposure to tumor cells and treatment was continued for 2 weeks afterward. Number of rats per group, *n* = 10 for all treatment groups except TAM (*n* = 15) and E_2_S1000 (*n* = 5).

### Tumor Measurement

Palpation of mammary tumors began 2 days post mammary tumor induction. BW was also monitored daily after development of measurable tumors. For tumor volume estimation, the longest and shortest diameters of the tumor were measured in all rats with a digital caliper.

Tumor volume was calculated using the formula: length × width^2^/2, as previously described by Ciomei et al. (2007).

At the end of the treatment period, rats were euthanized under anesthesia by cardiac puncture with a sterile syringe. Blood samples were collected in Z-serum clot activator tubes and centrifuged at 3500 rpm (10 min) for serum separation. Serum samples were stored at -20°C for biochemical analysis. Mammary tumors were excised and weighed. The liver, kidneys, spleen, and heart were collected, weighed, snap frozen in liquid nitrogen, and stored at -20°C. For histological evaluation, sections of liver and kidney tissues were preserved in 10% neutral-buffered formalin solution. Five micrometers of thick sections of paraffin-embedded tissues were stained with hematoxylin and eosin (H&E) and slides were examined under a light microscope.

### Measurements of Biochemical Parameters

Biomarkers for organ function [alkaline phosphatase (ALP), aspartate aminotransferase (AST), alanine aminotransferase (ALT), creatinine, urea, and albumin] and lipid profile (total cholesterol, HDL-cholesterol, and triglycerides) were determined in the serum using kits from Horiba ABX Pentra (Montpellier, France) and analyzed on Horiba ABX Pentra 400 analyzer (Horiba ABX, France) according to the manufacturer’s guidelines.

### Cell Lysate Preparation and Western Blot Analysis

Rat mammary tumor LA7 cells and monkey kidney fibroblast COS-1 cells obtained from ATCC, United States, were cultured in high glucose (DMEM supplemented with 10% FCS and 1% penicillin–streptomycin). The human MCF-7 BUS BC cell line (a kind gift from A. Soto, Tufts University, Boston, MA, United States) was cultured in high glucose DMEM supplemented with 5% (v/v) heat-inactivated (HI)-FCS (Sigma-Aldrich, South Africa) and 1% penicillin–streptomycin.

The cells were seeded at a density of 2.5 × 10^5^ cells into 6-well tissue culture plates and allowed to grow to 80–90% confluency. For cell lysis, 400 μL/well of lysis/loading buffer (40 mM Tris–HCl, pH 6.8, 2% β-mercaptoethanol, 8% glycerol, 2% SDS, and 0.04% bromophenol blue) was added. Cells were scraped and transferred to sterile microcentrifuge tubes and boiled at 96°C for 10 min. The cell lysates were resolved using a 10% SDS–PAGE gel and transferred onto a nitrocellulose membrane (Bio-Rad Laboratories, Inc.). The blots were blocked in Tris Buffer Solution-Tween 20 (TBS-T) containing 10% non-fat powdered milk for 90 min at room temperature. For immunodetection, the blots were probed with primary antibodies specific for ERα (HC-20; 1:1000; Santa Cruz Biotechnology), ERβ (ab3576; 1:1000, Abcam), and GAPDH (FL-335;1:3000; Santa Cruz Biotechnology) overnight at 4°C and subsequently incubated with goat anti-rabbit IgG horseradish peroxidase conjugated secondary antibody (SC-2030; 1:1000; Santa Cruz Biotechnology) for 90 min at room temperature. The proteins were visualized with the ECL enhanced chemiluminescence detection kit (Bio-Rad Laboratories, Inc.) and bands were scanned and quantified with the myECL imaging system (ThermoScientific, United States).

### Statistical Analysis

The data from the rat study were expressed as either mean ± SEM or median ± interquartile range (IQR). The commercial software program, GraphPad Prism Version 5 (GraphPad Software Inc., San Diego, CA, United States) was used for statistical analyses and graphs. One-way analysis of variance (ANOVA), followed by Dunnett’s *post hoc* test was used to test for significant differences at *P* < 0.05 between the groups.

## Results

### Phenolic Composition of SM6Met

The major phenolic compounds present in SM6Met were quantified using HPLC-DAD analysis (**Table 1**). The compounds were from diverse phenolic sub-groups such as: flavones (vicenin-2, scolymoside, and luteolin), flavanones (hesperidin and eriocitrin), xanthones (mangiferin and isomangiferin), dihydrochalcones (PDG and HDH), benzophenones (IMG and IDG), and phenolic acids (protocatechuic acid and *p*-coumaric acid). The newly prepared extract of SM6Met, used in the current study, contained all the compounds quantified in previously prepared SM6Met extracts at comparable concentrations, except for luteolin, which was present at a lower concentration than in previous extracts, and IMG and protocatechuic acid, which were present at higher concentrations than in previous extracts (Visser et al., 2013; Mortimer et al., 2015).

**Table 1:** Major polyphenols present in newly prepared *Cyclopia subternata* extract, SM6Met, as analyzed by HPLC-DAD.

Compound	Content (g/100 g SM6Met)
Scolymoside	2.844
Hesperidin	1.881
Mangiferin	1.844
3′,5′-Di-β-D-glucopyranosylphloretin (PDG)	1.776
3-Hydroxyphloretin-3′,5′-di-*C***-**hexoside (HDH)^∗^	1.370
3-β-D-Glucopyranosyl-4-β-	
D-glucopyranosyloxyiriflophenone (IDG)	0.999
Eriocitrin	0.820
3-β-D-Glucopyranosyliriflophenone (IMG)	0.775
Isomangiferin	0.553
Vicenin-2	0.187
Protocatechuic acid	0.154
Luteolin	0.023
*p*-Coumaric acid	co-elution

*^∗^Quantified in PDG equivalents.*

### The Decrease in the Growth of LA7-Induced Mammary Tumors Caused by SM6Met Is Comparable to That of the SOC, Tamoxifen

**FIGURE 2:**
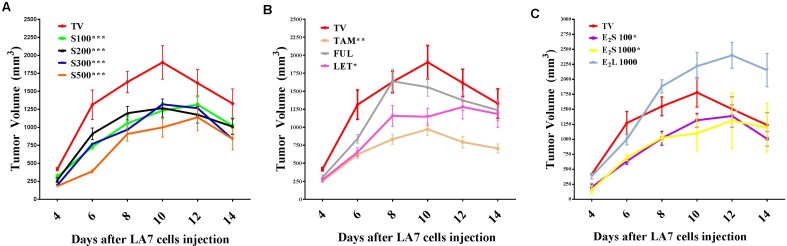
Time course of mammary tumor volume progression in treated rats. **(A)** SM6Met (S) at varying doses, **(B)** SOC therapies, and **(C)** E_2_. Points are indicative of average value of tumor volume ± SEM. Statistical analysis was performed using repeated measures ANOVA plus Dunnett’s *post hoc* test comparing the mean tumor volume time courses of treatments to vehicle-treated tumor control (TV). Tests were considered statistically significant at *P* < 0.05 (^∗^*P* < 0.05; ^∗∗^*P* < 0.01; ^∗∗∗^*P* < 0.001). See text for further details.

To investigate the effects of treatments on tumor growth in a rat model of mammary gland tumorigenesis, LA7 cancer cells (18 × 10^6^) were injected orthotopically into the mammary fat pad of female Sprague-Dawley rats for tumor induction. Although most of the rats developed palpable tumors as early as 48 h after injection of cells, measurable tumors were only developed in all the rats from day 4. Tumor incidence was one in all cases and thus only tumor volume and weight were compared. Tumors in the vehicle (TV) and water (TW)-treated groups showed a rapid growth progression from day 4 with a peak volume observed on day 10 (**Figure 2** and **Supplementary Figure S1**). Tumor volumes in vehicle- and water-treated groups were not statistically different, which shows that the vehicle did not significantly impact tumor growth. From day 11, tumor volume declined in these rats independent of treatment and by day 14, tumor volumes had declined considerably in the two control groups.

Daily oral administration of the well-characterized phytoestrogenic extract, SM6Met (**Table 1**) at four doses (100, 200, 300, and 500 mg/kg BW) for 28 days, starting 14 days prior to tumor induction, substantially reduced tumor volume relative to TV at all doses (**Figure 2A**). Specifically, the full-time course of tumor volume for all four SM6Met concentrations was significantly (*P* < 0.001) reduced compared to control (TV).

Of the SOC therapies investigated, TAM (10 mg/kg BW), a SERM, significantly (*P* < 0.01) decreased tumor volume progression and a substantial reduction in tumor volume was observed until day 14. Interestingly, TAM is the only SOC endocrine therapy that has previously been investigated on LA7-induced tumor growth and the inhibitory effect of TAM observed in the current study is in line with results of previous investigators (Shamsabadi et al., 2013; Karimian et al., 2015). This is the first study to report on the effects of the SOC therapies, FUL and LET, on LA7 mammary tumorigenesis (**Figure 2B** and **Supplementary Figure S2**). We did not observe a substantial effect on the time course of tumor volume progression after treatment with FUL, although a significant reduction (*P* < 0.01) in tumor growth on day 6 was recorded. In contrast, treatment of tumor-bearing rats with LET significantly (*P* < 0.05) reduced tumor growth from days 4–10 in comparison to TV, however, the effect was not as pronounced as for TAM.

Treatment with E_2_S100 and E_2_S1000 significantly (*P* < 0.05) suppressed tumor growth but only up to day 8. Furthermore, as shown in **Figure 2C**, statistical analysis of the full time course up to 14 days indicates that both the E_2_S100 and the E_2_S1000 groups are statistically different from the control group (TV). Moreover, in the E_2_L1000-treated rats, tumor volume increased substantially in comparison to TV (**Figure 2C**), although the increase in tumor volume was only significant (*P* < 0.05) from day 12 post tumor induction (after 26 days of E_2_ treatment).

The time course of percentage tumor inhibition (**Supplementary Figure S2D**) indicates that at the highest concentration (S500), the effect of SM6Met is not statistically different from the SOC, TAM, but does differ significantly from FUL (*P* < 0.001) and LET (*P* < 0.05). Specifically, the tumor-suppressing effect of SM6Met at the highest dose was comparable to that of TAM on day 4 post tumor induction (SM6Met; 56.1%, vs. TAM; 40.0%), day 6 (SM6Met; 70.30%, vs. TAM; 52.5%), day 8 (SM6Met; 44.3%, vs. TAM; 48.8%), and day 10 (SM6Met; 47.5%, vs. TAM; 48.9%). However, it should be noted that while SM6Met was introduced 14 days prior to tumor induction, TAM treatment only commenced after tumor induction.

### Effect of Treatments on Tumor Growth on Day 10 and Day 14 Post Tumor Induction

**FIGURE 3:**
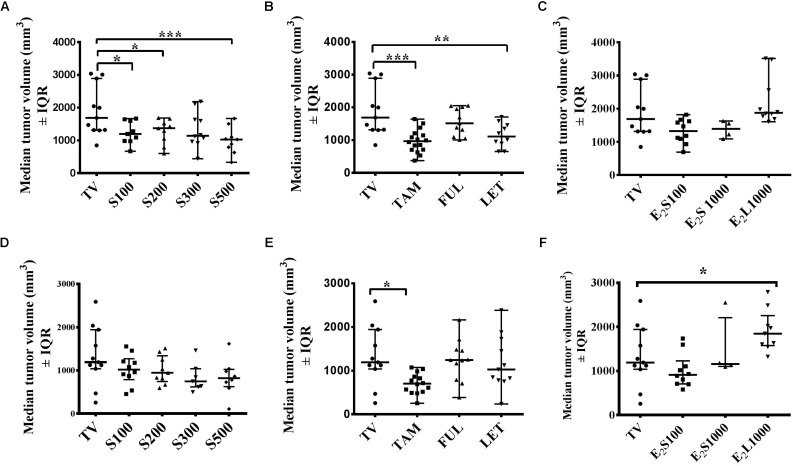
Effects of treatments on mammary median tumor volume on the 10th day **(A–C)** and 14th day **(D–F)** post tumor induction. **(A,D)** SM6Met, **(B,E)** SOC endocrine therapies, and **(C, F)** E_2_. Data are presented as median tumor volume with interquartile ranges (IQR). Statistical analysis was performed using one-way ANOVA plus Dunnett’s *post hoc* test comparing all treatments to vehicle-treated tumor control (TV). Tests were considered statistically significant at *P* < 0.05 (^∗^*P* < 0.05; ^∗∗^*P* < 0.01; ^∗∗∗^*P* < 0.001).

Although progressive tumor growth was observed in the tumor control (TV) group from day 4 to day 10, an initial decline in tumor volume was noted after day 10 in the TV control group, which continued to day 14 (**Figure 2**). Thus, a comparison at these 2 days seems warranted and **Figure 3** represents the effects of treatments on median tumor volume on day 10 and day 14 post tumor induction.

Although SM6Met significantly delayed tumor growth in rats compared to TV (**Figure 2A**), this effect was not observed beyond day 10. Thus while median tumor volume was significantly (*P* < 0.05) reduced in the SM6Met treatment groups on day 10, the decrease was not significant on day 14 (**Figures 3A,D**). However, rats treated with SM6Met still had a reduced median tumor volume on day 14 post tumor induction compared to TV control. Specifically, a reduction of 23.2, 24, 37, and 37%, respectively, for the S100, S200, S300, and S500 groups, was observed, while a 47% inhibition was observed with TAM. A direct statistical comparison between all the SM6Met groups and TAM on day 14 also indicates no significant difference.

In terms of the SOC therapies, interestingly, by the end of the treatment period (day 14), a significant reduction (*P* < 0.01) in median tumor volume was only sustained in TAM-treated rats in comparison to the TV group (**Figures 3B,E**). Although LET significantly (*P* < 0.01) suppressed median tumor volume on day 10 (**Figure 3B**), no significant suppression was observed on day 14 in groups treated with LET and FUL (**Figures 3B,E**).

The protective effect of E_2_ in the E_2_S100 and E_2_S1000 treatment groups was not observed on day 10 and day 14 (**Figures 3C,F**). However, exposure of rats to E_2_ before LA7 cell injection in the E_2_L1000 group significantly increased median tumor volume in rats on day 14 (*P* < 0.05), but not on day 10. These results suggest the influence of treatment duration and dose in the dual role of estrogen as a protector or a promoter of tumor growth.

### Effect of Treatments on Tumor Mass on Day of Euthanisia

**FIGURE 4:**
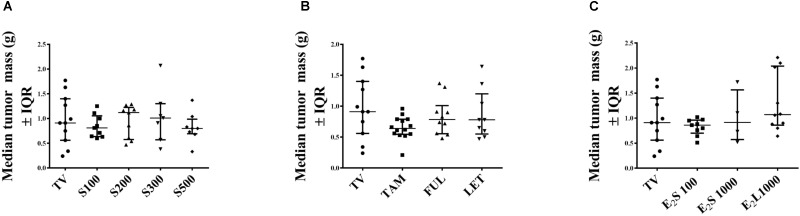
Effects of treatments on tumor mass (g) in rats on day of euthanasia. **(A)** SM6Met, **(B)** SOC endocrine therapies, and **(C)** E_2_. Data are presented as median ± IQR. Statistical analysis was performed using one-way ANOVA plus Dunnett’s *post hoc* test comparing tumor mass of treatments to vehicle-treated tumor control (TV). Tests were considered statistically significant at *P* < 0.05.

At necropsy (day 15 post tumor induction), no significant difference was observed in tumor weight in all treatment groups in comparison to vehicle-treated tumor-bearing animals (**Figures 4A–C**). This may be explained by the continuous and considerable decline in tumor volume in vehicle-treated TV starting from the 10th day post tumor induction (**Figure 2**).

### Effect of Treatments on Body Weight and Selected Organ Weights in LA7-Induced Tumorigenic Rats

As shown in **Table 2**, BW gain was found to be significantly lower (*P* < 0.05) in the TV group in comparison to non-tumorigenic rats treated with vehicle (CV). Furthermore, no significant difference was observed between TW and TV groups (**Table 2**), which indicates that it is rather the induction of the tumor and not use of vehicle that caused a significant reduction in BW in the TV group. No significant difference in BW gain was observed in rats administered SM6Met and SOC endocrine drugs (TAM, FUL, LET) in comparison to TV rats. However, significantly lower BW gain was observed in the S500 (*P* < 0.001) and TAM-treated rats (*P* < 0.001) relative to the CV group. Additionally, rats in the E_2_L1000 treatment group, but not the E_2_S1000 or E_2_S100 groups, had significantly (*P* < 0.05) lower BW gain compared to the TV group. Furthermore, BW in both the E_2_L1000 and E_2_S1000 groups differed significantly (*P* < 0.01) from the CV group.

**Table 2:** Effect of treatments on BW and selected organ weights.

	BW gain (g)	% Relative organ weight			
		Liver	Kidney	Heart	Spleen
CV	53.90 ± 3.03	3.79 ± 0.07	0.73 ± 0.01	0.38 ± 0.01	0.21 ± 0.01
TV	38.74 ± 3.51^b^*	3.82 ± 0.11	0.69 ± 0.01	0.38 ± 0.01	0.23 ± 0.01
TW	46.83 ± 5.80	3.43 ± 0.03^a^*	0.70 ± 0.02	0.36 ± 0.01	0.24 ± 0.01
S100	47.91 ± 4.95	3.74 ± 0.08	0.71 ± 0.02	0.38 ± 0.00	0.23 ± 0.01
S200	50.21 ± 4.61	3.62 ± 0.08	0.69 ± 0.02	0.37 ± 0.01	0.24 ± 0.01^b^*
S300	45.76 ± 4.87	3.81 ± 0.06	0.72 ± 0.01	0.37 ± 0.01	0.24 ± 0.01
S500	29.50 ± 1.95^b^***	3.52 ± 0.05	0.71 ± 0.01	0.38 ± 0.01	0.22 ± 0.01
TAM	31.10 ± 2.00^b^***	3.79 ± 0.10	0.74 ± 0.01	0.39 ± 0.01	0.23 ± 0.01
FUL	44.22 ± 4.73	3.47 ± 0.07^a^*	0.70 ± 0.01^b^*	0.38 ± 0.01	0.22 ± 0.01
LET	52.19 ± 2.86	3.59 ± 0.06	0.65 ± 0.01^b^**	0.37 ± 0.01	0.23 ± 0.01
E_2_S100	48.71 ± 3.39	3.63 ± 0.09	0.71 ± 0.01	0.38 ± 0.01	0.23 ± 0.01
E_2_S1000	28.52 ± 3.61^b^**	4.03 ± 0.11	0.72 ± 0.01	0.36 ± 0.01	0.19 ± 0.01
E_2_L1000	22.81 ± 1.94^a^*,b**	4.52 ± 0.06^a^***,b***	0.71 ± 0.01	0.36 ± 0.01	0.19 ± 0.00^a^**

*Data are presented as mean ± SEM. Statistical analysis was performed using one-way ANOVA plus Dunnett’s post hoc test. Tests were considered statistically significant at P < 0.05 (^∗^P < 0.05; ^∗∗^P < 0.01; ^∗∗∗^P < 0.001). ^a^Values differ significantly from those of vehicle-treated tumor control (TV). ^b^Values differ significantly from vehicle-treated normal control (CV). % Relative organ weight = (organ weight (g) on day of sacrifice)/(BW (g) on day of sacrifice) × 100.*

No significant alteration was observed in relative organ weights (%) after SM6Met and TAM treatment, when compared to TV and CV groups, except for a significant elevation (*P* < 0.05) in relative spleen weight (%) in the S200 group in comparison to CV group. Furthermore, LET did not significantly affect organ weights in comparison to TV, but did significantly (*P* < 0.001) reduce kidney weight in comparison to the CV group. FUL significantly (*P* < 0.05) reduced liver weight in rats, but did not alter the weight of the kidney, heart, and spleen, in comparison to TV treatment group. Treatments with FUL, however, significantly (*P* < 0.05) reduced kidney weights when compared to the CV group. No significant alteration was observed in organ weights in the two E_2_S treatment groups (E_2_S100 and E_2_S1000). A significant (*P* < 0.001) increase in liver and a significant (*P* < 0.01) decrease in spleen weights in the E_2_L1000 treatment group relative to TV were observed, even though no marked effect of treatment was observed on the % relative weights of kidney and heart.

### Effect of Treatments on Serum Biomarkers and Histological Changes of Organ Function and Lipid Profile

**FIGURE 5:**
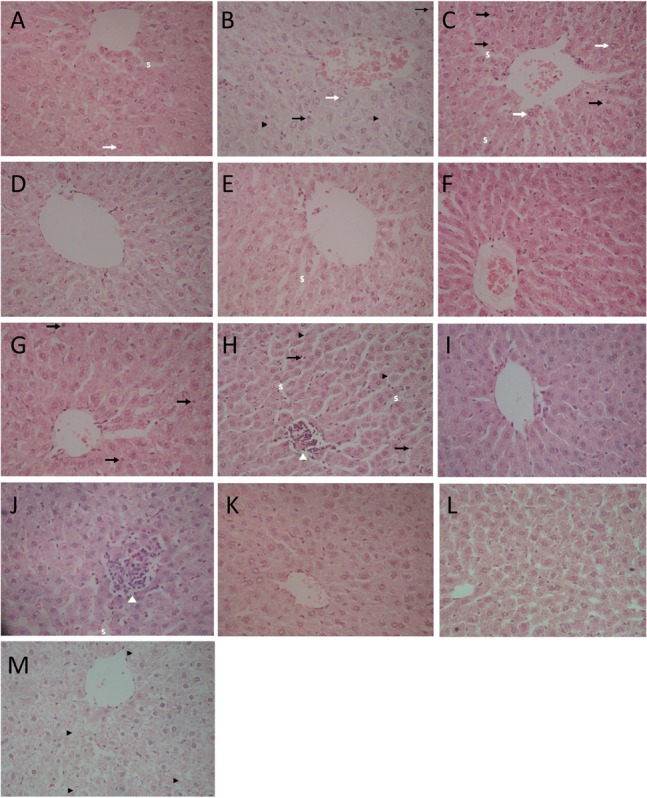
Light micrographs of liver sections stained with hematoxylin–eosin (HE; 400×). **(A)** CV group depicts slightly enlarged hepatocytes, vesicular nuclei (white arrows), and slightly compressed sinuses (S); **(B)** TV group shows slightly enlarged hepatocytes, vesicular nuclei (white arrows), pyknotic nucleus (black arrows), and single cell necrosis (black arrow heads); **(C)** TW group shows vesicular nuclei (white arrows), pyknotic nucleus (black arrows), dilated hepatic sinusoids (S); **(D)** SM100 group shows normal liver architecture; **(E)** SM200 group shows dilated sinusoids (S); **(F)** SM300 group shows enlarged hepatocytes; **(G)** SM500 group shows pyknotic nucleus (black arrows) and enlarged hepatocytes; **(H)** TAM group shows focus of lymphocyte infiltrate with loss of cellular details (white arrow head), pyknotic nuclei (black arrows), and single cell necrosis (black arrow head); **(I)** FUL group shows normal liver architecture; **(J)** LET group shows enlarged hepatocyte, dilated sinusoids (S) with lymphocyte infiltrate (white arrow head); **(K,L)** E_2_S (100 and 1000 μg/kg/day, respectively) groups showing normal liver architecture and slightly enlarged hepatocytes; **(M)** E_2_L1000 group reveals hepatocyte degeneration and necrosis (black arrow heads).

**FIGURE 6:**
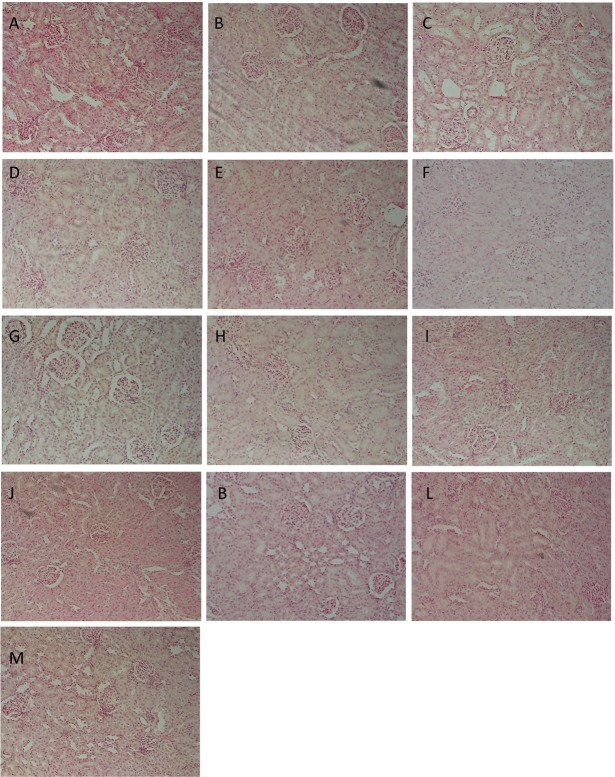
Representative histological photomicrographs of kidney sections stained with hematoxylin–eosin (HE; 400×). **(A)** CV; **(B)** TV; **(C)** TW; **(D–G)** SM6Met (100, 200, 300, and 500 mg/kg/day, respectively); **(I)** FUL; **(J)** LET; **(K,L)** E_2_S (100 and 1000 μg/kg/day, respectively); **(M)** E_2_L1000 showing normal structure of renal glomeruli and **(H)** TAM showing shrunken glomeruli.

**Table 3** represents the effects of the phytoestrogenic extract, SM6Met, E_2_, and the SOC endocrine drugs on biochemical parameters in rats. The increase in liver mass seen in TV group in comparison to TW, possibly due to castor oil, was not accompanied by a significant decrease in BW (**Table 2**) nor a significant increase in the levels of liver damage markers (**Table 3**), thus we regard the effect of vehicle treatment a non-adverse effect (Hall et al., 2012). Previous work (Irwin, 1992) that has shown that castor oil significantly increases liver weights and ALP levels also came to the same conclusion and ascribed the changes to increased metabolic activity rather than toxicity. As observed in the TV group, tumor induction caused a significant elevation of ALP (*P* < 0.01) in comparison to the CV group, whereas other serum markers of liver injury (ALT and AST) were unchanged. Liver injury in the TV group is supported by liver sections showing necrosis (**Figure 5**). Furthermore, no significant differences were observed in the levels of serum albumin, a marker of both liver and kidney damage (Clark and Kruse, 1990), lipid profile (total cholesterol, HDL-cholesterol, and triglycerides), and kidney function markers (urea and creatinine) in the TV group in comparison to the CV group. Micrographs of kidney sections showed normal structure for the TV group (**Figure 6**).

**Table 3:** Effect of treatments on biochemical parameters in the serum of normal and tumorigenic rats.

	ALP (U/L)	ALT(U/L)	AST (U/L)	Urea (mmol/L)	Creatinine (μmol/L)	Albumin (g/L)	T-CHOL (mmol/L)	HDL-CHOL (mmol/L)	Triglycerides (mmol/L)
CV	100.6 ± 4.3	41.7 ± 3.3	108.6 ± 4.7	7.1 ± 0.2	47.1 ± 1.0	33.6 ± 0.5	1.5 ± 0.1	0.7 ± 0.1	0.8 ± 0.1
TV	135.8 ± 6.3^b^**	38.0 ± 1.7	121.7 ± 6.8	7.3 ± 0.3	47.1 ± 1.5	31.0 ± 0.60	1.5 ± 0.0	0.7 ± 0.0	0.7 ± 0.1
TW	123.4 ± 3.1	33.1 ± 1.7	93.9 ± 11.37	6.3 ± 0.4	45.5 ± 4.0	30.8 ± 0.4	1.5 ± 0.1	0.7 ± 0.0	0.8 ± 0.0
S100	133.6 ± 10.2^b^*	45.9 ± 1.9	135.8 ± 11.0	6.2 ± 0.3	46.7 ± 2.2	30.9 ± 1.293	1.5 ± 0.1	0.7 ± 0.0	0.7 ± 0.0
S200	129.3 ± 4.9^b^*	35.2 ± 3.1	114.5 ± 12.3	6.4 ± 0.2	44.9 ± 1.2	30.8 ± 1.1	1.5 ± 0.1	0.7 ± 0.1	0.8 ± 0.1
S300	138.4 ± 6.9^b^**	38.3 ± 2.2	147.0 ± 18.3	7.0 ± 0.2	44.5 ± 3.0	30.7 ± 0.5	1.5 ± 0.1	0.7 ± 0.1	0.8 ± 0.1
S500	106.8 ± 8.180	40.1 ± 1.6	135.1 ± 12.9	7.2 ± 0.4	47.9 ± 0.8	32.1 ± 0.6	1.5 ± 0.0	0.7 ± 0.0	0.7 ± 0.1
TAM	192.6 ± 11.8^a^*** ,b***	43.1 ± 3.8	124.6 ± 13.2	8.3 ± 1.2^b^*	48.6 ± 1.9	32.0 ± 0.4	1.5 ± 0.1	0.8 ± 0.0	0.8 ± 0.1
FUL	127.6 ± 8.7^b^*	41.6 ± 2.8	132.9 ± 12.4	6.5 ± 0.4	46.2 ± 2.7	30.6 ± 1.4	1.3 ± 0.1	0.7 ± 0.0	0.8 ± 0.0
LET	145.1 ± 5.0^b^**	52.3 ± 3.0^a^**	171.2 ± 20.0^a^* ,b**	7.1 ± 0.1	41.6 ± 1.3	31.0 ± 0.1	1.5 ± 0.1	0.7 ± 0.0	0.5 ± 0.1
E_2_S100	130. 6 ± 9.8	30.0 ± 2.3^b^*	96.1 ± 9.7	5.8 ± 0.2^a^**,b*	43.0 ± 1.3	34.2 ± 0.9	1.3 ± 0.1	0.5 ± 0.1^a^***	0.7 ± 0.1
E_2_S1000	90.22 ± 4.4^a^**	40.6 ± 3.0	125.4 ± 4.3	7.3 ± 0.5	46.2 ± 0.8	29.2 ± 1.5^b^**	1.1 ± 0.1^a^* ,b*	0.4 ± 0.0^a^***,b**	1.8 ± 0.2^a^***
E_2_L1000	105.9 ± 6.1	61.3 ± 3.5^a^***,b***	131.3 ± 10.3	7.9 ± 0.4	45.5 ± 1.3	37.1 ± 0.9^a^*** ,b*	1.2 ± 0.0^a^* ,b**	0.5 ± 0.0^a^***	1.3 ± 0.2^a^***

*Data are presented as mean ± SEM. Statistical analysis was performed using ANOVA plus Dunnett’s post hoc test. Tests were considered statistically significant at P < 0.05 (^∗^P < 0.05; ^∗∗^P < 0.01; ^∗∗∗^P < 0.001). ^a^Values differ significantly from those of vehicle-treated TV. ^b^Values differ significantly from vehicle-treated normal control (CV). T-CHOL, total cholesterol; HDL-CHOL, HDL-cholesterol; U/L, units per liter; mmol/L, millimoles per liter; μmol/L, micromoles per liter; and g/L, grams per liter.*

SM6Met was non-toxic to the rats at all doses, as no significant alteration in the levels of the serum liver enzymes (ALT, AST, and ALP) was observed compared to TV. Furthermore, SM6Met did not significantly affect serum levels of albumin, urea, or creatinine. On the other hand, the result of our study showed a direct toxic effect of tumor induction (in TV group) and treatments (TAM, LET; dissolved in vehicle) on liver function evident by significant alterations in serum markers of liver injury, whereas these effects were not observed with SM6Met treatments. The histological effects observed with SM6Met in the liver, as not accompanied by significant alterations in enzyme markers in comparison to TV could therefore be due to vehicle’s effect or provoked by tumor induction. In addition, lipid profiles of tumorigenic rats treated with SM6Met did not differ from the TV or CV group.

Whereas no significant change was observed in all investigated biomarkers following FUL treatment, alteration in liver function markers was observed with the other SOC endocrine treatments, TAM and LET. Specifically, TAM significantly elevated serum ALP concentrations (*P* < 0.001), while LET increased AST and ALT levels in comparison to TV (**Table 3**). Lipid profile and serum levels of urea, creatinine, and albumin did not differ in all groups treated with the SOC endocrine drugs compared to TV. However, TAM significantly increased urea levels relative to the CV group. These results are supported by the kidney micrographs (**Figure 6**) showing kidney damage by TAM.

E_2_ treatment altered the serum lipid profile. In the E_2_S1000 and E_2_L1000 groups, the levels of total cholesterol and HDL-cholesterol were significantly (*P* < 0.001) reduced, while triglyceride levels were significantly (*P* < 0.001) elevated compared to the TV group. A significant (*P* < 0.01) reduction in the serum levels of HDL-cholesterol was also noticed in the E_2_S100 group, whereas levels of total cholesterol and triglycerides were not significantly altered in this group.

An extended treatment with E_2_ (E_2_L1000 group) markedly elevated (*P* < 0.001) serum ALT concentrations compared to TV, but no significant changes were observed with ALP and AST concentrations. Markers of liver injury were not elevated in the E_2_S groups, although a significant decrease in ALP and ALT levels were observed in E_2_S1000 (*P* < 0.01) and E_2_S100 (*P* < 0.05) groups, relative to the TV and CV groups, respectively.

Albumin levels were significantly elevated in E_2_L1000 group, but not the E_2_S1000 group, while creatinine and urea levels were not significantly affected in either group. The only clinical situation that causes an elevation in serum albumin is acute dehydration (Clark and Kruse, 1990). However, treatment with a lower dose of E_2_ (E_2_S100 group) significantly reduced (*P* < 0.01) serum urea levels. Furthermore, kidney sections (**Figures 6K–M**) suggest normal structure.

### Expression of Estrogen Receptor (ER) Subtypes in LA7 Cells

**FIGURE 7:**
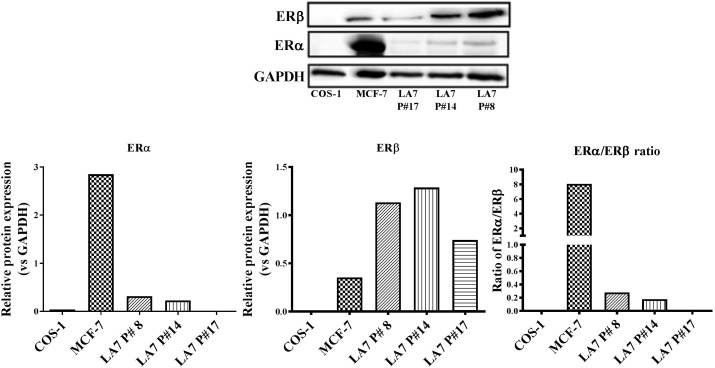
ER subtype expression in LA7 cells. A representative western blot is shown displaying the ER (ERα and ERβ) status of the rat mammary cell line (LA7), human BC cell line (MCF-7), and monkey kidney fibroblast (COS-1) cells. Quantification of the ER subtypes was done using the myECL Imaging software and is presented in figures below western blot.

Estrogen receptor expression is an important prognostic factor for BC and is vital for endocrine treatment recommendation. Thus, an investigation of the ER expression levels in LA7 cells is relevant to the current study. As a control, the expression of ERα and ERβ in the human BC cell line, MCF-7 (high ERα/ERβ ratio), and ER-negative monkey kidney fibroblast COS-1 cells were examined in the present study. As expected, ERα was more highly expressed than ERβ in MCF-7 cells, while no ER expression was detected in COS-1 cells (**Figure 7**). In contrast, ERα expression was weakly detected in LA7 cells, while ERβ was more highly expressed. The expression of both ER subtypes (ERα and ERβ) appears to decrease with increasing passaging of the LA7 cells. LA7 cells at passage number 14 (P#14), used for tumor induction in the present study, expressed a low ERα/ERβ ratio, which is indicative of higher ERβ levels compared to ERα levels.

## Discussion

Extended exposure to high levels of endogenous hormones, primarily estrogen, due to early menarche, late menopause, exogenous estrogen replacement therapy, prolonged oral contraceptive use, obesity, among others, is strongly associated with increased risk of BC (Travis and Key, 2003; Dall and Britt, 2017). The tumor promoting effects of E_2_ (Li et al., 2002; Singh et al., 2011) and synthetic estrogens (Palmer et al., 2002; Ueda et al., 2002; Singh et al., 2011) are also well documented in animal models of mammary carcinogenesis. Data from the current study, however, revealed that administration of E_2_ at 100 μg/kg BW/day (E_2_S100) and 1000 μg/kg BW/day (E_2_S1000) for 2 weeks significantly (*P* < 0.05) suppressed tumor progression (**Figure 2C**) in comparison to vehicle-treated controls, at least in the first 10 days post mammary tumor induction. In contrast, the higher dose of E_2_ (E_2_L1000) administered for a prolonged period (4 weeks) starting before tumor induction, significantly (*P* < 0.05) stimulated tumor progression at a later stage (26 days, i.e., 12 days post tumor induction). Although the observed tumor suppressive effect of estrogen in the current study contrasts with evidence supporting the tumor promoting effects of estrogen, this may be explained in part by the administered estrogen dose and treatment duration.

Although numerous studies have emphasized the primary role of estrogen in increasing BC risk, the inhibitory role of E_2_ (20 μg) injected concomitantly with 4 mg progesterone on mammary cancer over a 40-day period has been reported (Grubbs et al., 1983). In addition, a sustained treatment with 20 μg E_2_ in silastic capsules for 1 week was effective in decreasing the multiplicity and increasing the latency of mammary tumorigenesis induced by MNU in rats (Rajkumar et al., 2001). Treatment with high dose estriol (30 mg, 200 μg) or ethinyl estradiol (100 μg), singly or in combination with progesterone for 1 or 3 weeks, also reduced mammary tumor incidence and multiplicity in a chemical carcinogen rat mammary model (Rajkumar et al., 2004). Furthermore, Rajkumar et al. (2007) demonstrated a protective effect of pregnancy estrogen levels against mammary tumorigenesis attained by a sustained exposure to 100 μg of E_2_, either alone or combined with progesterone, for 2 weeks in genetically engineered mouse models. Experimental evidence also exists for the protective action of short-term treatment (3 weeks or less) with physiological levels of E_2_ and progesterone against mammary carcinogenesis (Sivaraman et al., 1998; Guzman et al., 1999). Although the exact mechanisms involved in protection of BC by E_2_ are unknown, reductions in the proliferative potential of the mammary cells or hormone-induced changes at the mammary stroma or systemic level are some suggested mechanisms (Rajkumar et al., 2007). As macrophage infiltration and increased expression of inflammatory cytokines in the mammary tissues are characteristics of LA7-induced mammary tumors (Bakar et al., 2017), the immunomodulatory effect of exogenously administered estrogen (Attanasio et al., 2002; Lang, 2004) could also contribute to the delay in the tumor progression in this model. Nonetheless, our study is the first to report on the effect of E_2_ on tumor growth induced by LA7 cells.

The E_2_ dose (100 μg/kg BW/day or 1000 μg/kg BW/day) used in our study may be considered physiologically relevant. Administration of 100 μg, 200 μg, or 30 mg of E_2_ in silastic capsules in a previous study resulted in physiologically relevant E_2_ levels of 67, 94, and 143 pg/mL, respectively. These concentrations reflect the levels of serum E_2_ (55–630 pg/mL) in pregnant Long Evans and Sprague-Dawley rats (de Lauzon et al., 1974; Watson et al., 1975; Rajkumar et al., 2001). Furthermore, serum E_2_ levels corresponding to the mid-menstrual cycle in pre-menopausal women are between 100 and 400 pg/mL (Kratz et al., 2004). Although the oral route (hence first pass through the liver) of E_2_ administration in the present study, in addition to the influence of ovarian estrogen production, might result in differential E_2_ pharmacokinetics.

The data showed that treatment with SM6Met significantly delayed mammary tumor growth at all doses demonstrating its chemopreventative effect. The autoregressive nature of the tumor is observed in all the tested groups, including the TV group, and thus seems to be a property of the tumor model rather than the treatment. The inference on the chemopreventive effect of SM6Met was therefore based on the positive outcome of SM6Met treatment in retarding tumor growth in the first 10 days. Although this effect was not as profound after day 10, this does not invalidate the effect observed till day 10. Furthermore, as LA7 tumors contain a heterogeneous population of cells, ranging from stem cells to fully differentiated cells, that have proliferative capabilities that range from highly to limited proliferative capacity (Zucchi et al., 2007, 2008), the autoregression of the tumor observed after day 10 may be due to differentiation of the LA7 cells and thus it would appear that SM6Met treatment targets the highly proliferative undifferentiated cells in the tumor.

Although no obvious dose response was noticed with SM6Met treatment, the chemopreventative effect of SM6Met at the highest dose (500 mg/kg BW/day) was significantly higher (*P* < 0.01) than at the lowest dose (100 mg/kg BW/day) and was comparable to that of TAM. However, it should be noted that TAM, like other SOC’s (LET and FUL), was only administered after tumor induction to improve tolerability and minimize side effects. The hepatotoxic effect of TAM at a dose of 6 mg/kg administered over 2 weeks has been demonstrated in rats (Gao et al., 2016). The use of LET and FUL have been solely approved for BC treatment, while TAM is also widely use in treatment due to its limiting side effect profile as a chemopreventive agent. In contrast, administration of SM6Met prior to tumor induction was considered a more rational option in the present study as the leaves of the *C. subternata* from which SM6Met was extracted is used to produce a honey-flavored herbal infusion, widely consumed as honeybush tea in South Africa. Furthermore, since this is the first study to assess the potential toxic effect of SM6Met, a longer-term safety and tolerability assessment was considered warranted. Moreover, prevention of BC presumes prior administration of the drug or extract as evidenced in both animal (Hollingsworth et al., 1998; Wang and Zhang, 2017) and in clinical (Kinsinger et al., 2002) chemopreventative studies. However, as the LA7 model is an orthotopic method of tumor development, in terms of chemoprevention, it does not evaluate effects on cancer initiation or promotion. Furthermore, the current study, which focusses on secondary prevention (De Flora et al., 2001), cannot distinguish between the chemopreventative effects of SM6Met on tumor establishment and progression.

The inhibitory action of SM6Met on BC cell proliferation and cell cycle progression has been reported previously (Visser et al., 2013). More recently, in an MNU model of BC, rats fed a SM6Met formulated diet (containing 2500 ppm of the SM6Met aimed at an exposure of 200 mg/kg BW) for 140 days showed a significant reduction in tumor volume (53%) (Visser et al., 2016). In the present model of LA7-induced tumorigenesis, SM6Met treatment at an equivalent dose of 200 mg/kg BW (S200), but administered for only 26 days, reduced tumor volume by 33%, while a 48% reduction in tumor volume was observed at the highest dose (S500).

The chemopreventive effects of SM6Met may be attributable to the active compounds in the extract (**Table 1**). Some major phenolic compounds identified in SM6Met, particularly hesperidin (a flavanone) and mangiferin (a xanthone), have demonstrated anti-cancer effects *in vitro* (Lee et al., 2010; Cuccioloni et al., 2016). Specifically, mangiferin, the major xanthone in SM6Met, has demonstrated broad-spectrum efficacy against an array of different cancers *in vitro* and *in vivo* with an antitumor efficacy comparable to that of the anticancer drug, cisplatin (Gold-Smith et al., 2016). The structural requirements for the inhibitory effect of flavones on aromatase are attributed to the position of the 4′-hydroxyphenol group, which increases binding affinity to the aromatase enzyme (Kao et al., 1998; Rice and Whitehead, 2006). However, no study has specifically reported on the effect of scolymoside and vicenin-2 on BC, although these flavones contain a 4′-hydroxyphenol group and are thus potentially worthy of investigation for their AI and tumor-suppressing action. Although the cytotoxic and apoptotic effects of dihydrochalcones, specifically phloretin, have been reported in prostate cancer and breast tumor cells (Szliszka et al., 2010; Kim et al., 2013), to the best of our knowledge, there is no information on the antitumor effects of phloretin derivatives such as PDG and HDH, that have been identified in SM6Met (**Table 1**). Thus, it is likely that a complex interaction of these compounds is responsible for the antitumor effect of SM6Met via diverse mechanisms of action.

Furthermore, previous studies from our laboratory using BC cell lines have demonstrated the antiproliferative effects of SM6Met *via* a mechanism involving the selective ER subtype modulating (SERSM) action of SM6Met, by specifically demonstrating that SM6Met acts as an ERβ agonist and an ERα antagonist (Visser et al., 2013). The mammary tumor suppressive effects of SM6Met in the present study might thus be due in part, to its opposing effects on the ER subtypes.

An investigation of the ERα and ERβ expression status could provide insight into the possible mechanisms of action for pharmacological regulators of ER activity. In the present study, ERα, in contrast to ERβ, was weakly expressed in LA7 cells at all passages examined. However, western blot analysis also revealed a decrease in the ratio of ERα:ERβ protein expression with increasing cell passage number, highlighting the possible role of culturing conditions in receptor protein expression. The LA7 cells (P#14) used for tumor induction in the present study appeared to express higher ERα and ERβ protein levels compared to cells at a latter passage (P#17), which had no detectable ERα. An opposite ER subtype phenotypic profile (eightfold higher ERα than ERβ protein expression) was observed in the MCF-7 ER^+^ cells. Recently, in the study of Vela and Escrich (2016), no detectable ERα protein expression was observed in LA7 cells; however, there was no report on the protein expression status of ERβ.

Although it is well established that ERα promotes the growth of breast tumors, the specific functions of ERβ are less well understood. Experimental evidence exists for the inhibitory role of ERβ on the proliferative response of ERα in cells where they are co-expressed. ERβ expression has also been linked to a reduction in the aggressiveness of breast tumors and improved disease-free survival rates, compared with ERβ-negative tumors (Hartman et al., 2006).

The reason for an earlier tumor appearance and earlier regression in the current study in contrast the available evidence on growth progression of LA7-tumors (Syam et al., 2016) is not clear. Tumor progression was reported in the tumor control group for 4 weeks, while in our study, tumor growth was observed for only 10 days in the vehicle treated tumor control group (TV). Furthermore, we also detected measurable tumors as early as on the 4th day post tumor induction, in contrast to tumor detection at a later time-point (7–10 days) in this study. One could argue that the discrepancy between the two studies might be attributable to the higher number of cells (18 × 10^6^) used for tumor induction in the present study in comparison to 6 × 10^6^ cells used in the study of Syam et al. (2016). However, none of the other studies on LA7 tumor model reported tumor progression, ER subtype phenotype of the LA7 cells, and the passage number was not mentioned, therefore no comparison can be made in this regard.

Tamoxifen is the only SOC endocrine therapy that has previously been investigated in LA7 tumorigenesis. The observation from the current study of the suppressive effect of TAM on the growth of LA7 mammary tumorigenesis is in line with other studies (Shamsabadi et al., 2013; Karimian et al., 2015). Although the antitumor effect of TAM in the present study could be mediated via inhibition of ERα activation, the weak expression of ERα in the LA7 cells used in the current study, however, suggests the possible involvement of non-ER-mediated mechanisms of TAM action. Although TAM and its metabolite 4-hydroxy-TAM act by antagonizing the activation of ERα (Jordan, 2003; Bekele et al., 2016), the beneficial effects of TAM in BC patients with very low, or no ERα, have also been reported (Manna and Holz, 2016). Furthermore, numerous *in-vitro* and *in-vivo* BC models have reported on some ER-independent mechanisms of TAM action (Bogush et al., 2012).

In pre-menopausal hormone-responsive BC, AIs are not used clinically as a monotherapy because AIs reduce the negative feedback on the hypothalamic–pituitary–ovarian axis, further increasing ovarian estrogen production. Estrogen then competitively antagonizes binding of the inhibitor to the enzyme’s active site and thus overcomes the effects of AIs on the aromatase enzyme. The animal model utilized in the current study simulates pre-menopausal BC due to the presence of ovarian function and thus it is reasonable to infer that estrogen levels may be higher in LET-treated rats in comparison to tumor controls. The delay in mammary tumor growth in LET-treated rats in comparison to vehicle controls might thus be mediated by elevation of serum estrogen levels. However, blockade of ovarian estrogen production has been reported in rats at a high dose (5 mg/kg BW/day) of LET for 4 weeks (Sinha et al., 1998). On the other hand, some experimental evidence exists for the beneficial effects of AIs, like LET (Kubatka et al., 2008a) and anastrozole (Kubatka et al., 2008b), in a MNU-induced pre-menopausal mammary carcinogenesis model. Furthermore, Madeira et al. (2013) also documented a decrease in the proliferation marker, Ki67, in tumors of patients with much lower ERα than ERβ scores following treatment with anastrozole, another AI, suggesting a possible relationship between therapeutic response to AIs and ER subtype expression.

Data from this study revealed that oral treatment with FUL at 5 mg/kg BW/week did not significantly affect mammary tumor growth (**Figure 2** and **Supplementary Figure S2**). Although FUL has shown a moderate therapeutic effect against triple-negative BC (ERα^-^/ERβ^+^) and inhibition of hormone responsive (ERα^+^/ERβ^+^) BC via upregulation of ERβ mRNA and protein expression levels (Mishra et al., 2016), the lack of a significant effect on the time course of tumor growth after cyclic FUL regimens (oral administration of 5 mg/kg BW/week) in the present study could be due to the poor oral bioavailability of FUL (Robertson, 2007). A sustained exposure to FUL via an alternative route of administration should be considered in future studies.

Induction of mammary tumors in rats caused a drastic reduction in BW gain (**Table 2**). Another prominent outcome of this study is that SM6Met administration did not significantly affect BW gain in comparison to TV, which may be a possible reflection of an unaltered nutritional status in SM6Met-treated rats. No significant changes in BW gain in the TAM group were observed in comparison to TV, although a decreased weight gain in comparison to the vehicle-treated normal control (CV) group was statistically significant (*P* < 0.001). Furthermore, no significant alteration in BW gain was observed with any of the other SOC endocrine therapies (FUL and LET) in comparison to TV. The inhibitory effect of estradiol on food intake has been previously established (Blaustein and Wade, 1976; Eckel, 2004). This anorexic effect of estrogen is further supported by the prevention of weight loss in ovariectomized animals (Sasayama et al., 2017). A significantly reduced BW gain observed in the E_2_L1000 treatment group relative to TV group in our study could be partly due to this anorexic effect of E_2_.

The goal of cancer therapy is to promote cancer cell death, while minimizing or preventing toxicity to normal cells. No significant alterations in tissue weight was observed in the SM6Met-, TAM’-, and LET-treated rats in comparison to the TV treatment group, although a significant reduction (*P* < 0.05) in liver weight was seen with FUL treatment. The liver plays an important role in the metabolism of estrogens (Mitchel et al., 1976) and in the current study, a significant (*P* < 0.001) E_2_-induced liver enlargement was observed at the higher dose and longer treatment duration of E_2_ (E_2_L1000), implying a dose- and time-dependent hepatotoxic action of E_2_. The excessive accumulation of estrogen in the liver could interfere with secretion of other molecules from the liver parenchyma (Radzikowska et al., 2012), while the increased liver size might be an indication of drug-induced toxicity as reflected by the data obtained from analysis of liver injury markers.

The actions of E_2_ on the immune system are complex and differ according to its concentration and targeted functions (Straub, 2007; Khan and Ansar Ahmed, 2016). Estrogens suppressed spleen weight gain under inflammatory immunological conditions, but not in naive female mice (Zhou et al., 2011), suggesting the differential effects of estrogen under different immunological conditions. Mammary tumors induced by the orthotopic injection of LA7 cells are linked to increased activity of inflammatory mediators (Bakar et al., 2017) and thus in the current study, an elevation (*P* < 0.01) in relative spleen weight was noted in rats treated with E_2_ for a prolonged period (E_2_L1000).

Elevated serum concentrations of transaminases (AST and ALT) and ALP are diagnostic markers for liver injury (Giannini et al., 2005). It is noteworthy that SM6Met did not significantly raise the serum concentrations of liver biomarkers in comparison to the tumor control (TV) group demonstrating the maintenance of liver integrity in the SM6Met-treated groups. However, data from this study revealed a differential pattern in the alteration of serum enzymes in the TV, TAM, LET, and E_2_ groups suggesting different mechanisms of liver injury. The pattern of serum enzyme elevations may indicate the site of liver injury and distinguish between hepatocellular and cholestatic liver disease (Murali and Carey, 2014).

Alkaline phosphatase is a membrane-bound enzyme and is used for the assessment of plasma membrane integrity (Sharma et al., 2014). It is found on the surface of bile duct epithelia and may be elevated if bile excretion is inhibited, resulting in the accumulation of toxic bile acids that cause cholestatic liver injury (Limdi and Hyde, 2003). Specifically, in the current study, the preferential increase in serum ALP concentrations in the LA7-induced tumorigenic control rats (TV), which was significantly aggravated by TAM treatment, may therefore indicate the possibility of membrane damage and interference with bile excretory mechanisms in the liver. Micrographs for the TV and TAM groups also show some degenerative changes and infiltration of inflammatory cells. Data from the present study conform to that of a previous study demonstrating a serum upsurge of ALP in LA7-induced mammary tumorigenesis (Abbasalipourkabir et al., 2010). In contrast, serum concentrations of ALP were lowered in the E_2_ groups, although significant only in the E_2_S1000 group (*P* < 0.01), suggesting a hepatoprotective effect of E_2_, supported by micrographs showing normal liver architecture in this treatment group.

Alanine aminotransferase and AST are found abundantly in the cytosol of the hepatocyte and serum increases in these enzymes are associated with hepatocellular injury and increased membrane permeability (Solter, 2005). Elevation in serum levels of ALT after E_2_ treatment for 4 weeks in the E_2_L1000 group suggests damage to liver cells. In contrast, the reduction in ALT and AST levels in the E_2_S100 group demonstrates the hepatoprotective effect of E_2_. Collectively, the results suggest a dose-dependent hepatotoxic effect of E_2_. Furthermore, the significant elevation in the serum levels of ALT (*P* < 0.01) and AST (*P* < 0.05) by LET in the current study suggests drug-induced hepatocellular injury. FUL treatment, however, did not alter the levels of liver injury biomarkers in comparison to TV group.

Mammary tumor induction did not significantly affect the lipid profile in the TV group relative to the CV group. Furthermore, treatment with the phytoestrogenic extract, SM6Met, did not significantly alter lipid profiles nor did the SOC endocrine therapies. However, alteration in serum lipid profile was observed with E_2_ treatment (**Table 3**). The mechanism of estrogen-induced hypertriglyceridemia involves the promotion of hepatic triglyceride synthesis and a reduction in the activity of hepatic triglyceride lipase (Lee and Goldberg, 2008). Although the effects of exogenous estrogens on the lipid profile are dependent on the dose, route of administration, and formulation (Masood et al., 2010), they are generally characterized by a decrease in total and low-density-lipoprotein cholesterol and an increase in high-density-lipoprotein cholesterol and triglycerides (Kavanagh et al., 2009). Furthermore, the results from the current study are in line with the report on the cholesterol lowering effect of estrogen in ovary intact female rats (Weinstein et al., 1986). Contrary to clinical studies demonstrating the elevation of HDL-cholesterol by estrogens, the reduction in serum concentration of HDL-cholesterol in the present study may be due to species differences between rats and humans. In the rat, 60% of the circulating cholesterol is found in HDL and 30% in LDL, as opposed to the 30% found in HDL and 60% found in LDL in the human (Gwynne et al., 1976). The fact that HDL-cholesterol is the main serum lipoprotein cholesterol in rats may explain the significant reduction of HDL-cholesterol observed with E_2_ treatment in the current study.

## Conclusion

The LA7 mammary tumor model is a relatively new orthotopic method of tumor development that has the advantage of inducing mammary tumor in a much shorter time than chemically induced mammary tumor models (MNU and DMBA). The current study is the first to report on the effects of E_2_ and SOC endocrine drugs, such as FUL and LET, on the growth of LA7-induced mammary tumors. Paradoxically, the protective effects of short-term E_2_ treatment and endocrine therapy observed on tumor growth in this model may occur via different mechanisms of action (Rajkumar et al., 2007) and might indicate some level of E_2_ responsiveness. It might thus prove useful to evaluate the effects of potential antitumor agents using ovariectomized rats in future studies.

The tumor-suppressing effect of SM6Met in this study is remarkable considering the highly tumorigenic and aggressive growth of LA7-induced mammary tumors (Abbasalipourkabir et al., 2010). Moreover, the chemopreventative action of SM6Met is coupled to a significantly better safety profile than seen with the SOC endocrine therapies and highlights its promising potential for BC chemoprevention. Future studies will explore the underlying mechanisms by which SM6Met delay tumor growth.

## Author Contributions

OO, AK, and AL conceived and designed the experiments. OO, AK, NV, and DdB performed the experiments. MS and TM prepared and calibrated the ABX Pentra Automated Analyzer. OO, AL, and DdB analyzed the data. OO and AL drafted the manuscript. All authors critically revised the article for intellectual content and approved the final version to be published.

## Conflict of Interest Statement

The authors declare that the research was conducted in the absence of any commercial or financial relationships that could be construed as a potential conflict of interest. The reviewer WG declared a shared affiliation, with no collaboration, with the authors MS and TM to the handling Editor.

## Supplementary Material

FIGURE S1 Time-course of mammary tumor progression in rats treated with water (TW) or vehicle (TV). Points are indicative of mean tumor volume ± SEM. Number of rats in TW (*n* = 8) and TV (*n* = 10). Statistical analysis was performed using a paired two-tailed *t*-test and no significant difference was observed.

FIGURE S2 Time-course of percentage tumor inhibition (%TI). **(A)** SM6Met, **(B)** SOC endocrine therapies (TAM, FUL, and LET), **(C)** E_2_, and **(D)** a comparison of S500 with SOCs. Points are indicative of mean %TI. Statistical analysis for **(D)** was performed using repeated measures ANOVA plus Dunnett’s *post hoc* test comparing mean %TI time-course of all SOC treatments to S500. %TI time-course of S500 was not significantly different from that of TAM but was significantly different from that of FUL (*P* < ^∗∗∗^) and LET (*P* < ^∗^). Percentage tumor inhibition (%TI) was calculated as: (*Vc* – *Vt*)/*Vc* × 100 (de Sousa et al., 2007), where Vc and Vt are the mean tumor volumes of the negative control (group 3: TV) and treatment groups (groups 4–13), respectively.
