# Supplementary figures and images for: Chemoprevention of LA7-Induced Mammary Tumor Growth by SM6Met, a Well-Characterized Cyclopia Extract
Source: Front Pharmacol. 2018 Jun 20;9:650. doi: 10.3389/fphar.2018.00650 (PMC6019492; doi:10.3389/fphar.2018.00650)

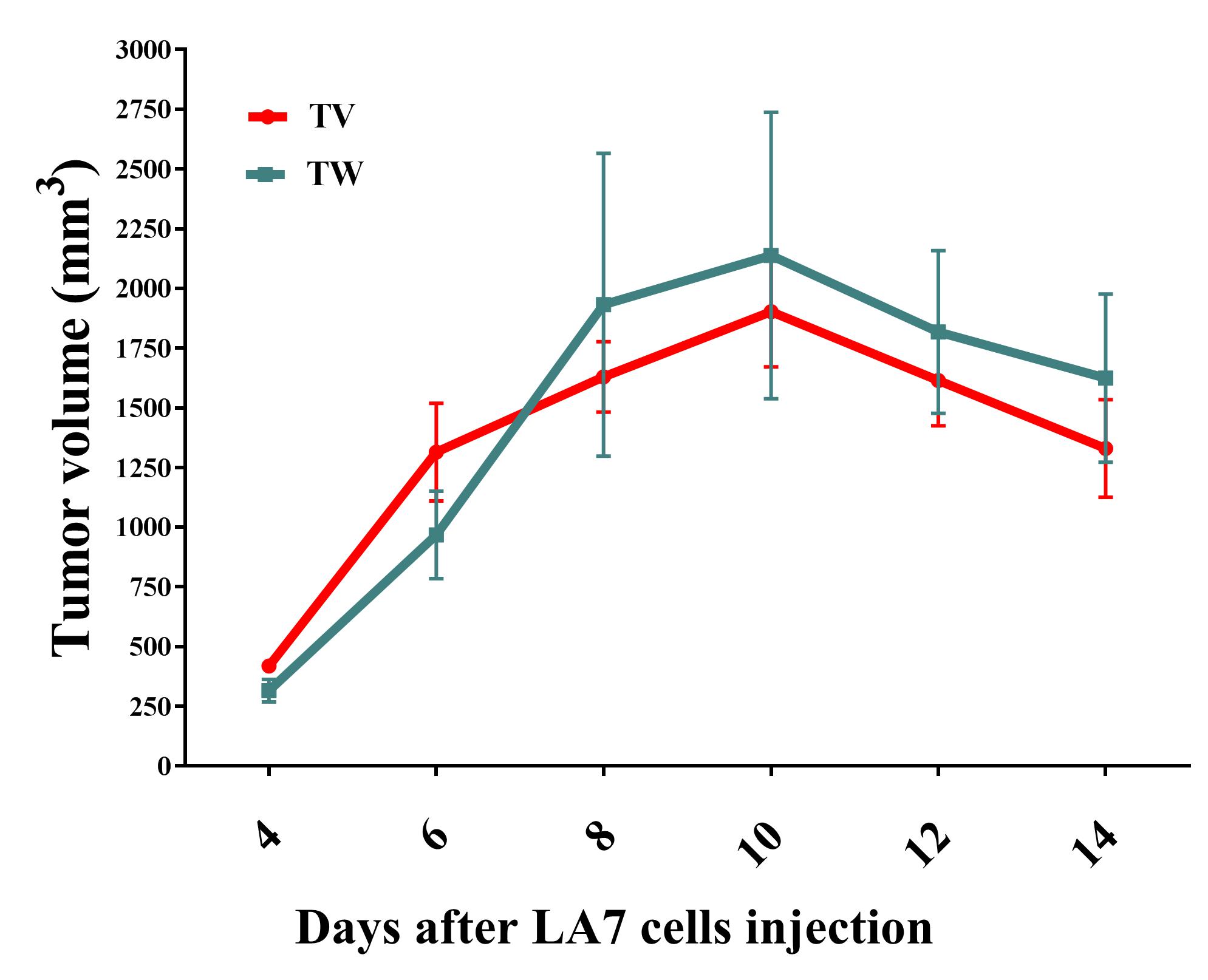

Supplement: FIGURE S1 — Time-course of mammary tumor progression in rats treated with water (TW) or vehicle (TV). Points are indicative of mean tumor volume ± SEM. Number of rats in TW (n = 8) and TV (n = 10). Statistical analysis was performed using a paired two-tailed t-test and no significant difference was observed. [file Image_1.JPEG]

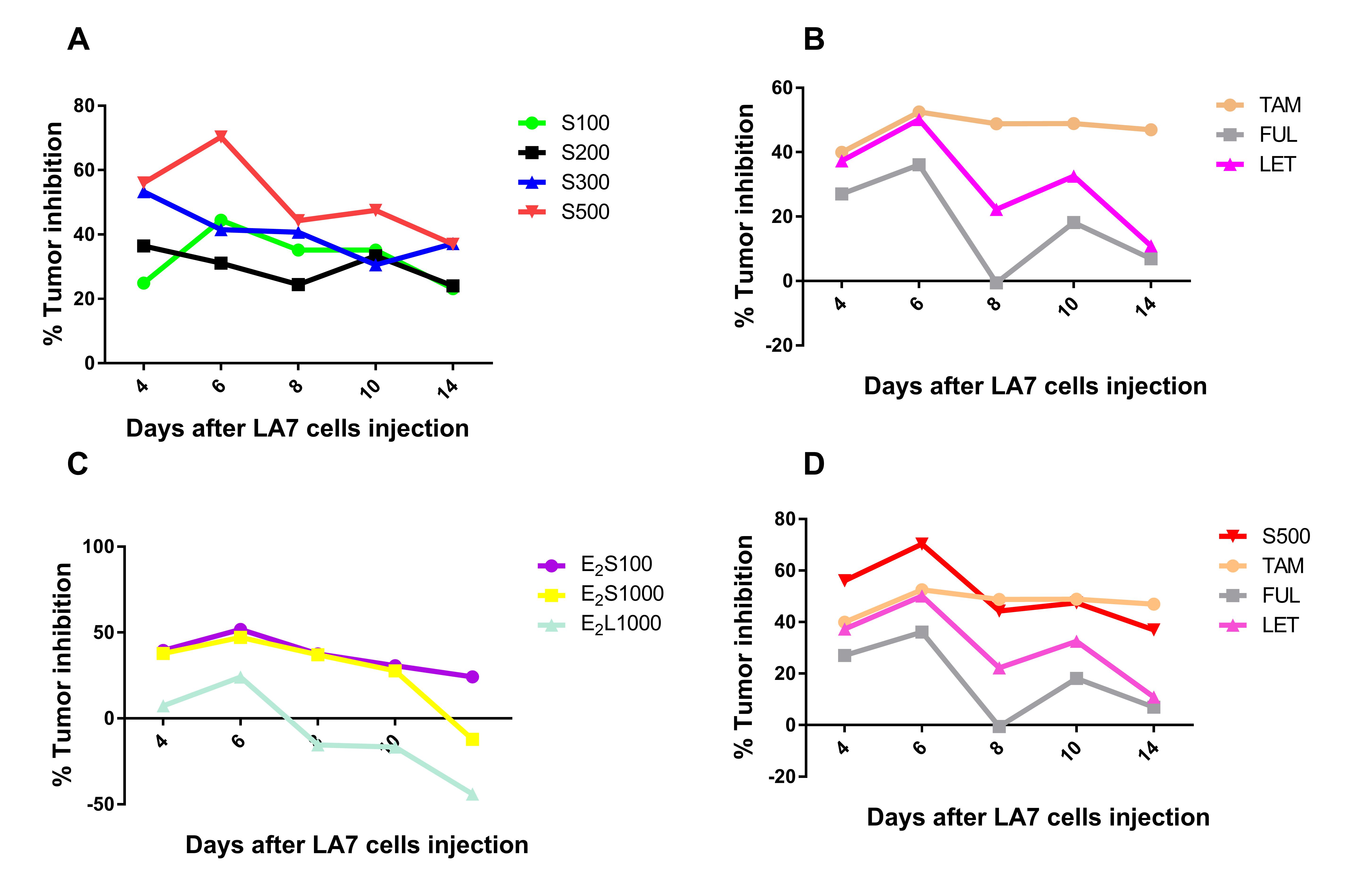

Supplement: FIGURE S2 — Time-course of percentage tumor inhibition (%TI). (A) SM6Met, (B) SOC endocrine therapies (TAM, FUL, and LET), (C) E2, and (D) a comparison of S500 with SOCs. Points are indicative of mean %TI. Statistical analysis for (D) was performed using repeated measures ANOVA plus Dunnett’s post hoc test comparing mean %TI time-course of all SOC treatments to S500. %TI time-course of S500 was not significantly different from that of TAM but was significantly different from that of FUL (P < ∗∗∗) and LET (P < ∗). Percentage tumor inhibition (%TI) was calculated as: (Vc – Vt)/Vc × 100 (de Sousa et al., 2007), where Vc and Vt are the mean tumor volumes of the negative control (group 3: TV) and treatment groups (groups 4–13), respectively. [file Image_2.JPEG]
